# 1368. In-hospital and post-discharge mortality among adults with COVID-19 associated hospitalization, COVID-NET, March 2020–April 2021.

**DOI:** 10.1093/ofid/ofad500.1205

**Published:** 2023-11-27

**Authors:** Kadam Patel, Pam Daily Kirley, Breanna Kawasaki, James Meek, Kyle P Openo, Maya Monroe, Justin Henderson, Ruth Lynfield, Daniel M Sosin, Adam Rowe, Sophrena Bushey, Eli Shiltz, Melissa Sutton, H Keipp Talbot, Ashley Swain, Michael Whitaker, Christopher Taylor, Fiona P Havers

**Affiliations:** Centers for Disease Control and Prevention, Atlanta, Georgia; California Emerging Infections Program, Oakland, California; Colorado Department of Public Health and Enviornment, Denver, Colorado; Connecticut Emerging Infections Program, Yale School of Public Health, New Haven, Connecticut; Georgia Emerging Infections Program and Atlanta VA Medical Center, Decatur, GA; Maryland Department of Health, Baltimore, Maryland; Michigan Department of Health and Human Services, Lansing, Michigan; Minnesota Department of Health, St. Paul, MN; New Mexico Department of Health, Santa Fe, NewMexico; New York State Department of Health, Albany, New York; University of Rochester School of Medicine and Dentistry, Rochester, New York; Ohio Department of Health, Columbus, Ohio; Oregon Health Authority, Portland, Oregon; Vanderbilt University Medical Center, Nashville, Tennessee; Salt Lake County Health Department, Salt Lake City, Utah; CDC, Marietta, Georgia; CDC, Marietta, Georgia; CDC, Marietta, Georgia

## Abstract

**Background:**

Since the COVID-19 pandemic began in 2020, > 1 million persons have died in the US due to COVID-19. While various research illustrated in-hospital mortality in detail, post-discharge mortality has not been well described. Data from the COVID-19-Associated Hospitalization Surveillance Network (COVID-NET) were used to describe in-hospital and post-discharge mortality among adults hospitalized with COVID-19 in the US during the first year of the pandemic.

**Methods:**

COVID-NET is a population-based surveillance system in 14 states that captures laboratory-confirmed COVID-19-associated hospitalizations, defined as any patient residing in the catchment areas with a positive SARS-CoV-2 test during hospitalization or ≤ 14 days prior to admission. Detailed chart reviews were conducted by trained surveillance officers on a representative sample stratified by age group, state, and month. Cases were linked to death certificate data to identify those who died ≤ 60 days after hospital discharge. Data were restricted to adults ages ≥ 18 years admitted from March 2020–April 2021. Proportions of mortality and hospitalization rate (per 100,000) were calculated by month to investigate trends in adults aged ≥ 75 years.

**Results:**

176,044 hospitalized adults met the COVID-NET case definition during the study period; 26,149 underwent full chart review. Overall, 9.7% died in-hospital and 6% died post-discharge; among those ≥ 75 years, these proportions were 18.1% and 16.2%, respectively (Figure 1). Among adults who died post-discharge, 47.5%, 33.1% and 28.3 were discharged to another facility, hospice, and private residence +/- services, respectively. Post-discharge mortality among those ≥ 75 years exceeded in-hospital mortality during October 2020 and December 2020 – February 2021. Hospitalization rates among those ≥ 75 years peaked in December 2020; the following month, January 2021, had the highest proportion of post-discharge mortality (22.6%) in this age group (Figure 2).

Mortality among hospitalized adults ages ≥18 years with laboratory-confirmed COVID-19, COVID-19-Associated Hospitalization Surveillance Network, March 2020–April 2021

**Figure:**
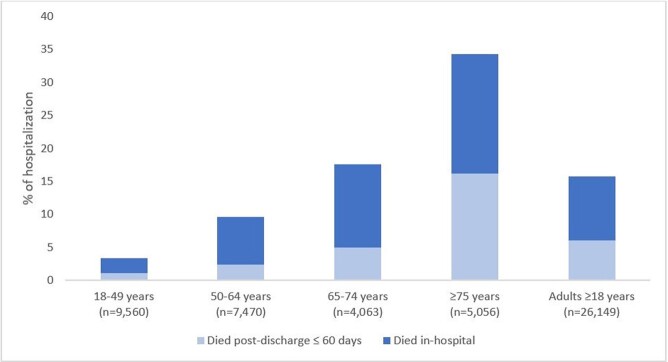

**Figure 1**

Hospitalization Rate and Mortality among laboratory-confirmed COVID-19 among adults ages ≥75 years, COVID-19 Associated Hospitalization Surveillance Network, March 2020–April 2021

**Figure:**
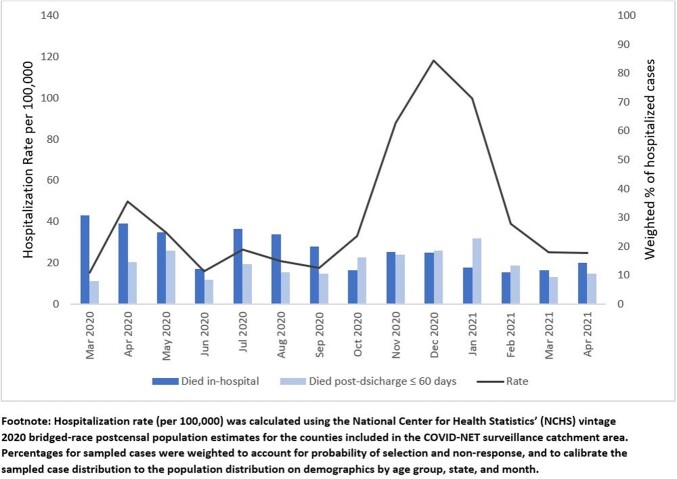

**Figure 2**

**Conclusion:**

Among all deaths in adults with a COVID-19-associated hospitalization, two-thirds occurred in-hospital and one-third occurred ≤ 60 days post-discharge. Increases in post-discharge mortality followed a period of high COVID-19-associated hospitalization rates among ≥ 75 years.

**Disclosures:**

**All Authors**: No reported disclosures

